# A Combined Telemedicine and Ambulatory Wound Care Team Intervention for Improving Cross-Sector Outpatient Chronic Wound Management: Protocol for the Mixed Methods TELE-AMBUS Research Project

**DOI:** 10.2196/55502

**Published:** 2024-11-04

**Authors:** Sindre Aske Høyland, Kari Anne Holte, Olaug Øygarden, Kamrul Islam, Egil Kjerstad, Ragnhild Gjerstad-Sørensen, Synnøve Aske Høyland, Hanne Rusten Wærnes, Pascale Carayon, Maureen Fallon, Sarah Bradbury, Marcus Gürgen, Sissel Eikeland Husebø, Eirin Rødseth

**Affiliations:** 1 Division for Health and Social Sciences Norwegian Research Centre Stavanger Norway; 2 Department for Dermatology Stavanger University Hospital Stavanger Norway; 3 Department for Industrial and Systems Engineering University of Wisconsin-Madison Madison, WI United States; 4 Welsh Wound Innovation Centre Ynysmaerdy Wales United Kingdom; 5 Department of General Practice Institute of Health and Society University of Oslo Oslo Norway; 6 Research Group of Nursing and Healthcare Sciences Stavanger University Hospital Stavanger Norway; 7 Faculty of Health Sciences University of Stavanger Stavanger Norway; 8 Department for Personal E-Health Norwegian Centre for E-health Research Tromsø Norway

**Keywords:** chronic wound management, specialist health care sector, primary health care sector, ambulatory wound care team, telemedicine, process evaluation, economic evaluation, observations, interviews, whole system framework

## Abstract

**Background:**

There is a growing prevalence of nonhealing wounds and chronic diseases in society, and there is an associated need for wound management solutions that include the use of telemedicine. A broad spectrum of factors influences the planning and execution of interventions within telemedicine in chronic wound management, spanning organizations, technologies, and individuals, including professionals and patients. The Telemedicine and Ambulatory Wound Care Team (TELE-AMBUS) project applies a whole-system research approach to account for this spectrum of factors.

**Objective:**

The primary objective of this study was to explore and analyze the implementation and consequences of an outpatient wound management model, comprising 2 interconnected quality improvement interventions (ie, telemedicine and ambulatory wound care team) aimed at older and vulnerable patients with chronic wounds, across the specialist and primary health care sectors. Embedded in this objective is the aim to improve the competence levels of health care providers and, consequently, the service quality of outpatient wound management across specialist and primary health care services.

**Methods:**

This project examines the implementation and consequences of an outpatient wound management model through a combined process and economic evaluation research strategy. A sociotechnical system theory approach and multiple work package design support the examination. The project uses observations, conversations, interviews, and economic assessments to gather rich, in-depth insights and understanding on why and how the new wound management model contributes to a change or not compared with the traditional treatment model.

**Results:**

The project has been funded from 2021 to 2025. Baseline interviews have been conducted since April 2022 and concluded in January 2024. Fieldwork, including nonparticipant observations, semistructured interviews, and informal conversations, has been conducted since November 2022 and is expected to conclude in March 2025. In parallel and as part of the cost-effectiveness analyses, time usage data on the outpatient and regular clinical models are being gathered during the fieldwork.

**Conclusions:**

We applied a whole-system approach in multiple ways, that is, to design or inform our fieldwork and to explore, evaluate, and translate project findings into practice across services. To our knowledge, this approach has not been undertaken in telemedicine in chronic wound management literature and associated human factors and ergonomics research. Thus, our approach can produce both original and novel research and theoretical results internationally.

**International Registered Report Identifier (IRRID):**

DERR1-10.2196/55502

## Introduction

### Background

The prevalence of nonhealing wounds (ie, not healing within 6 weeks) and chronic diseases in society is growing, which impacts both patients’ quality of life and the costs of health care delivery [1-3]. Furthermore, society is facing an increasingly overburdened and financially strained health care system due to global pandemics, multifront conflicts and wars, economic decline, and so forth. To alleviate the overall burden on the health care system, the Norwegian National Health and Hospital Plan 2020-2023 focuses on integrated health care services that are closer to patients’ homes. The plan emphasizes the creation of outreach hospitals that will provide more health care in patients’ homes; collaborate better with municipal health and care services, both in person and online; and work more closely with other hospitals [4]. Aligning with this plan, the Norwegian Digital Strategy for the Public Sector 2019-2025 highlights that public services shall be perceived as seamless and integrated by the users [5]. Reflecting the concern for chronic states in society and associated political measures, as manifested in national health planning, this article presents the protocol for the research project “TELE-AMBUS – Exploring and redesigning a cross-sector outpatient chronic wound management model comprising telemedicine and ambulatory wound care interventions.” The Telemedicine and Ambulatory Wound Care Team (TELE-AMBUS) project was initiated based on (1) a strong desire of our project partner, the Wound Diagnostic Centre (WDC) in Stavanger, to test an outpatient chronic wound management model, combined with (2) identified knowledge needs within the domain of telemedicine in chronic wound management interventions.

Regarding current domain knowledge, our systematic literature review revealed a need for broader or more comprehensive empirical exploration into quality improvement and integration of telemedicine and chronic wound management [6]. These explorations should expand on and extend beyond the currently identified intervention barriers and opportunities. Specifically, barriers of telemedicine in chronic wound management include delays in installing the telemedicine software and workforce shortages, which, over time, leads to a steep decline in individuals using telemedicine [7]; limitations set by the organization and technology in terms of management focus, resources, economy, consultation time, wound training, and the need for updated equipment [8,9]; and qualities of the individual or professional such as the patient’s confidence in the competence and professional skills of health professional [10]. On the flip side, opportunities generated by telemedicine in chronic wound management include the telemedicine technology’s potential for improving wound evaluation accuracy by means of tools and software developments [11-14]; the potential that gathering individual health care professionals with a shared focus on the patient can enhance clinical outcomes across the wound spectrum, clinical care settings, and geographical locations [15]; and the health-related quality of life dimension that professional, patient, and next-of-kin express satisfaction with telemedicine as a treatment method [14,16]. Shedding light on both quality of life and monetary cost reduction opportunities, Dardari et al [17] found that “...telemedical intervention with an expert nurse could lead to a length of hospitalization and direct costs that are two times lower compared to conventional follow-up.”

Concerning the specific outpatient model explored in the TELE-AMBUS project, wound specialist nurses at the WDC expressed a years-long desire to explore an outpatient model where a team of specialist nurses, in consultation with the relevant hospital physician, provides ambulatory wound care diagnosis and treatment to vulnerable patients with chronic, nonhealing wounds that have various mobility challenges and reside in the municipalities. The mobility challenges include the patient being older or frail, having advanced or complex wounds, and experiencing travel stress and appointment difficulties. The underlying rationales for testing the model are that (1) patients may be evaluated by nursing and physician specialists before their conditions deteriorate; (2) vulnerable patients’ quality of life may improve as the pain and discomfort caused by wounds is reduced; (3) primary care (municipal) health care professionals may learn from the specialist ambulatory wound care team (AWCT) and be provided with a more direct line of communication with the hospital specialists through telemedicine, thus improving general wound care for patients; and (4) the AWCT provides early interventions aided by the telemedicine solution that may reduce costs at the hospital and primary care facilities.

Illustrated by the contextual backdrop of national plans and strategies, the current knowledge status, and the outpatient wound management model, intervention barriers and opportunities have as much to do with the organizations and the individuals involved as with the technology. Thus, research conducted on interventions within telemedicine in chronic wound management needs to consider a broad spectrum of factors that can influence the planning and execution of interventions with factors spanning organizations, technologies, and individuals, including professionals and patients. This spectrum of factors or conditions has been accounted for in the TELE-AMBUS project’s whole system research approach (described in the *Methods* section). Our approach resonates with Kahn’s [18] suggestion:

Researchers should explore the crucial issue of context, studying not only whether telemedicine [and by extension, the overall outpatient model in this project] works but also how, when, and where it works best, to provide a roadmap for more effective implementation.

### Key Definitions

Telemedicine, as a central concept in the outpatient model of the TELE-AMBUS project, can be clarified as the use of electronic information and communication technology to exchange health care information between health care practitioners across sites and distances, which can improve health care delivery and outcomes including patients’ health status [19]. The telemedicine solution selected for the TELE-AMBUS project involves the primary care nurses using a secure cell phone to send wound pictures to the specialist AWCT, who reviews and logs the pictures (through the patient’s journal) and responds to the primary care nurses with suggestions and recommendations. This is not a dedicated telemedicine solution that is currently unavailable in Norway. Telemedicine is part of eHealth, which is understood as health care services provided electronically through the internet.

The specialist AWCT is a team of specialized wound nurses who travel to the patient’s home, examine and treat the patient, and educate the present municipal health personnel on the wound treatment procedure applied to the patient.

### Primary and Secondary Project Objectives

Targeting the knowledge needs identified above, as well as the WDC’s desire to test an outpatient chronic wound management model, the TELE-AMBUS research project has the primary objective: to explore and analyze the implementation and consequences of an outpatient model comprised of 2 interconnected quality improvement interventions (ie, telemedicine and AWCT) aimed at older and vulnerable patients with chronic wounds across the specialist and primary health care sectors. Embedded in this objective is the aim to improve the competence levels of health care providers and, consequently, service quality of outpatient wound management (diagnosis and treatment) across specialist and primary health care services.

In addition, four secondary objectives support and extend the primary objective, grounded in qualitative and quantitative approaches: (1) to systematically review and synthesize existing knowledge on interventions within telemedicine in chronic wound management, including barriers and opportunities and associated measures or elevation efforts across the specialist and primary health care sectors (described in the *Introduction* section and in Høyland et al [6]); (2) from a sociotechnical system (STS) theory approach focused on the Systems Engineering Initiative for Patient Safety (SEIPS) model, to explore and analyze the outpatient model or interventions and cross-sector work system (discussed in the *Methods* section); (3) from a health economic perspective, to assess the cost-effectiveness of implementing an outpatient model across the specialist and primary health care sectors; and (4) to transform project findings into managerial and employee strategies and practices across specialist and primary health care. Each secondary objective constitutes an individual work package (WP) in the TELE-AMBUS project.

## Methods

### Setting and Participant Recruitment

The WDC, responsible for organizing and running the new outpatient wound intervention, is located at the Department of Dermatology, Stavanger University Hospital, Norway. Stavanger University Hospital employs more than 7600 individuals and provides health care services to 18 municipalities with a population of about 350,000. The WDC is unique in a Norwegian context, as specialists from different professions and disciplines (wound specialist nurses, a vascular surgeon, and a dermatologist) gather to examine patients in one location comprehensively and assemble coherent treatment plans. The WDC operates each Tuesday with cross-disciplinary wound assessment and treatment. Each week, the WDC has an average of 2 patients with diabetic wounds, 1-2 patients with arterial wounds, 3 patients with venous wounds, 2 patients with immunological wounds, and 1 patient with a pressure wound, totaling 10 patients. The size of the ulcerations varies from the size of a pin to most of the leg. Healing varies according to wound size; the patient’s condition (physical and mental); activity levels and self-insight; the competency levels of municipal health personnel, including wound follow-up; and more.

There are other interdisciplinary wound centers comparable to the WDC in Norway in terms of being interdisciplinary and outpatient-focused and sharing publicly financed health care systems and institutions. Within the Nordic countries, there are the Copenhagen Wound Healing Centre at Bispebjerg University Hospital (Denmark), the University Center of Wound Healing in Odense (Denmark), and the Wound Center (Haavakeskus) at Helsinki University Hospital (Finland). Extending further, the former Welsh Wound Innovation Centre in the United Kingdom is focused on outpatient chronic wound healing and innovation.

The project manager from the Norwegian Research Centre, in dialogue with the WDC, conducted the initial municipal partner recruitment process as well as subsequent calls for additions or adjustments of included subunits (home care services and nursing homes), with the latter process being facilitated by the project manager’s contact with appointed coordinators in each municipality. The WDC’s main criterion for selecting municipalities was practical proximity to the hospital running the outpatient intervention, specifically, about 1-hour driving distance. In addition to a regional university hospital in Norway, a total of 6 municipalities, varying in size, chose to participate in the research project. Of these, 5 of the municipalities included home care services and nursing homes, while 1 municipality limited inclusion to nursing homes and only 1 of 5 home care services zones. Regarding recruitment of patient cases for the intervention, the AWCT at the WDC receives and processes ongoing patient referrals from units in the municipalities. Specifically, the wound specialist nurses or AWCT receive patient referrals from general practitioners in the municipalities. The AWCT assesses whether the referred patient is a suitable candidate for the intervention, with the selection criteria being hard-to-heal wounds and the patient having a multimorbid condition. The exclusion criteria are immunological and diabetic wounds. Both the WDC and municipalities self-organize the internal recruitment of study participants for research activities in the TELE-AMBUS project.

To gain empirical insights into the process of implementing and operating the outpatient intervention, combining AWCT and telemedicine implementation and operation, individuals involved with the intervention will be asked to participate in research activities, including observations (ie, being observed by the researchers), semistructured interviews, and informal conversations. Combining observations with asking questions, including clarification questions, constitutes a contextual design approach [20]. To account for both service ends of the intervention, invited participants will include the AWCT, which was comprised of 2 wound specialist nurses and the nursing staff in the municipalities (from home care services and nursing homes). In addition, key informants associated with the outpatient intervention will be invited to participate in semistructured interviews, including health care managers, information technology personnel, and other stakeholders at different levels of the hospital system. Table 1 provides an overview of the participants involved in the project’s intervention and research activities.

**Table 1:** Categories and types of participants involved in the intervention and research activities in the Telemedicine and Ambulatory Wound Care Team (TELE-AMBUS) project, including regional, national, and international partners.

Project participant category	Project participant type	Specific roles
Regional partner: university hospital	Wound specialist nurses, a dermatologist, a vascular surgeon, a general surgeon, information technology–personnel, higher- and mid-level managers, including the local project coordinator and economy department	Operated the new wound management model; participated in observations, conversations, and interviews; participated in regular scientific advisory meetings
Regional partners: 6 municipalities	Medical and nonmedical trained nursing staff at home care services and nursing homes; higher- and mid-level managers at the municipal level, including local project coordinator and economy department	Participated in observations, conversations, and interviews; one municipal partner participated in regular internal meetings at the university hospital
National partner: Norwegian Centre for E-health Research	Senior advisor	Participated in regular scientific advisory meetings, partner-specific meetings, and dissemination activities
International partners: WWIC^a^ and the University of Wisconsin-Madison	WWIC: chief operating officer, clinical research director, professor in chronic wounds; University of Wisconsin-Madison: professor in system theories	Participated in regular scientific advisory meetings, partner-specific meetings, and dissemination activities

^a^WWIC: Welsh Wound Innovation Centre.

To summarize, the inclusion criteria applied by the TELE-AMBUS project were (1) municipalities with practical proximity to the hospital running the outpatient intervention, (2) vulnerable patients with chronic, nonhealing wounds who have various mobility challenges and reside in the municipalities (discussed in the *Introduction* section), and (3) all health care professionals and levels involved in the AWCT intervention (Table 1). In addition, the study applied the following exclusion criterion: patients with diabetic foot ulcers who belong to another hospital department and are not involved in the outpatient intervention.

Given that we study a hospital-initiated intervention where patient visits (cases) are generated by referrals from the involved municipalities, no sample size limitation was defined. However, we have recently crossed the threshold of 30 patient visits or cases, which includes 30 observations (by 1-2 participating researchers) and associated on-site and later follow-up interviews with municipal health personnel and the AWCT. Our impressions of the newer patient visits are beginning to converge with impressions from previous visits, suggesting the effect of data saturation. However, due to the ongoing nature of this intervention and our desire to pursue “challenging” cases (where things go less according to plan), of which we have fewer, we expect that further patient visits will be conducted until the first quarter of 2025 and perhaps beyond (depending on perceived data saturation), likely ending with around a total of approximately 40 patient visits or cases.

### Research Approach

#### Overview

Improvement interventions, defined broadly as purposeful efforts to secure positive change, have become an increasingly important focus of activity within health care, including wound management [21-23]. The larger intervention studied in the TELE-AMBUS project is the outpatient wound management model itself, which can be considered an improvement intervention in its initial form improvement rather than research directed [21] due to the wound specialist nurses’ ownership and desire to explore the model in the context of older and vulnerable patients. It is also a complex coordinated care intervention, comprising multiple interacting and intertwined components (telemedicine and AWCT) across different levels and organizations, in this case, involving primary and specialist health care services, and in the case of primary health care, numerous municipalities with specific characteristics [15]. Therefore, the most appropriate design is process evaluation because it can “assess fidelity and quality of implementation, clarify causal mechanisms and identify contextual factors associated with variation in outcomes” [24]. Supportive of this design, we identified a need for broader or more comprehensive empirical explorations into quality improvement and integration of telemedicine in chronic wound management to capture the complexity of organization, technology, and individual interplay across sectors (discussed in the *Introduction* section).

#### The Process Evaluation

For the TELE-AMBUS project, we use a qualitative longitudinal study design, following and analyzing the intervention as it unfolds in practice. We apply the process evaluation framework by Moore et al [25], with descriptions of the intervention, implementation, mechanisms, and outcomes in relation to the context, and where we use several data collection and analysis methods. The qualitative research approach comprises situated methods and data collection that are carried out for a long period of time (for this project, the systematic literature review, nonparticipant observations, semistructured interviews, and informal conversations are situated and carried out in an outpatient wound management setting for a long period of time); a view that the social world is multifaceted and thus requires a certain degree of interpretation (outpatient chronic wound care occurs in a complex setting of multiple disciplines and technologies, types of patients, and so forth across sectors); and an inherent exploratory nature suited to the study and conceptualization of new or emerging phenomena, such as this project’s tandem exploration of telemedicine and AWCT [26-31].

Findings of our systematic literature review on the topics of telemedicine and ambulatory wound care management interventions [6] shape a basic mental orientation for the fieldwork, including the observational protocol and associated observations of the AWCT and nursing staff during patient consultations. This mental orientation also assists in identifying the broader and more specific questions to raise during informal conversations and in-depth semistructured interviews with managers and employees across specialist and primary health care case organizations. Seminars and digital meetings with project partners conducted throughout the project period, where partner insights and experiences (including from specialists in wound management, innovation, eHealth, health economy, and system perspectives) and empirical findings are presented, serve to adjust and “sharpen our field lens” in terms of observational and interview focus (such as, what barriers or outcomes to explore further) for the continuing fieldwork. Furthermore, the lens sharpening effect is also reversed, whereby we as researchers apply our whole system perspective in partner meetings and dialogue as well as during the intervention (through observations, interviews, and conversations), thus challenging the partners to adopt a broader understanding of systems, organizations, and change.

#### The Economic Evaluation

Regarding the quantitative approach applied in the project, we aim to conduct a health economic evaluation of the outpatient model (intervention) compared with the regular clinical wound management model. An economic evaluation will measure 2 parameters, costs and outcomes or effects, and compare the 2 approaches. Intended to inform decision-making, the idea behind an economic evaluation is not necessarily to identify which of the 2 improvement interventions is the “better” one; rather, if the cheapest option is the most effective, it will be the most cost-effective or dominant option, or if the cheapest option is not the most effective, incremental cost-effectiveness ratio will be measured [32]. There are several methods for the economic evaluation of health care programs. In the context of this project, it is relevant to focus on the intervention. Since the new model or intervention will also have consequences for resource allocation, considerations of both cost and benefits (here, health improvement) are necessary and fundamental to quality improvement evaluation (eg, the Institute for Healthcare Improvement, United States). There are alternative perspectives on the economic evaluation of health care programs as well. In this project, we are concerned with a health care sector perspective, that is, focusing on consequences for primary and specialist health care and the patient. Potentially, the intervention may lead to both short-term and longer-term health improvements. Short-term improvements may follow from earlier referrals to specialist health care, thereby resulting in less severe admissions to the hospital, shorter length of stay, less pain and discomfort for the patients, and potentially quicker recovery. In the longer term, increasing competence and awareness in primary health care services can potentially reduce the number of severe cases admitted to the hospital. Specific measures and outcomes of health improvements are in the short-term analyses related to positive changes in the number of hospital admissions due to a reduction in serious and complicated cases compared with the regular wound model. In the longer term, health improvements are related to positive changes in the number of admitted wound patients, regardless of whether they are complicated or not, compared with the regular wound model. Costs may change, too, for both primary and specialist health care services, and the relative distribution of costs between services may change. The regular wound model too often seems to result in severe and complicated admissions to the hospital, a situation that increases costs both at primary care and specialist care levels. However, one consequence of a suboptimal level of competence and capacity at the primary health care level is that some of the treatment costs are passed on to specialist health care. Hospitals are compensated for at least part of these costs through the remuneration system (diagnosis-related groups–based), but that is not a relevant issue from a societal or health care sector perspective.

The economic evaluation will be based on case data collected during the intervention period. The data include reported time usage by nurses and physicians, allowing cost calculations to be made in Norwegian Krone. The intervention’s costs, including equipment costs, are compared with treatment as usual costs collected from participating municipalities. Based on relevant *International Classification of Diseases, Tenth Revision* (*ICD-10*) codes, register data from the Norwegian Patient Register are used to estimate nationwide costs associated with wound treatment. To the extent the intervention contributes to reducing the use of hospital care and hospital resources for the treatment, we can estimate net benefits and model the potential aggregate net benefits nationwide of the intervention.

Summarized, the TELE-AMBUS project applies a combined process and economic evaluation research strategy with associated qualitative and quantitative endpoints that can affect the primary and specialist health care systems within and across. The end points include improvements in cost-effectiveness (economic evaluation) and competence and service quality (process evaluation) of the new outpatient model versus the regular clinical wound management model.

#### STS Approach

Reflecting the knowledge limitations identified in the *Introduction* section, our conceptual and analytical whole system approach selected for this project is based on system theories. STS theory concerns interactions between and joint design of the technological subsystem, the personnel subsystem, and relevant external surroundings, being mutually interdependent and where any alterations or changes in one part of a system (eg, technology such as telemedicine) will impact other parts of the system [33]. Embedded in the STS tradition is the human factors and ergonomics (HFE) discipline focusing on systems where humans interact with the environment “to jointly improve performance and well-being by designing the integrative whole better, and by integrating the human into the system better” [34]. The core characteristics of the HFE discipline are, therefore, (1) the system approach, (2) the design-driven approach, and (3) the focus on outcomes related to both system performance and well-being [34]. Perspectives and models from the HFE discipline are recommended in the *World Health Organization’s Global Patient Safety Action Plan* [35], specifically, the second strategic objective to “build high-reliability systems and health organizations that protect patients daily from harm,” that is, the emphasis is on designing safe and sustainable health care systems for the future.

**Figure 1:**
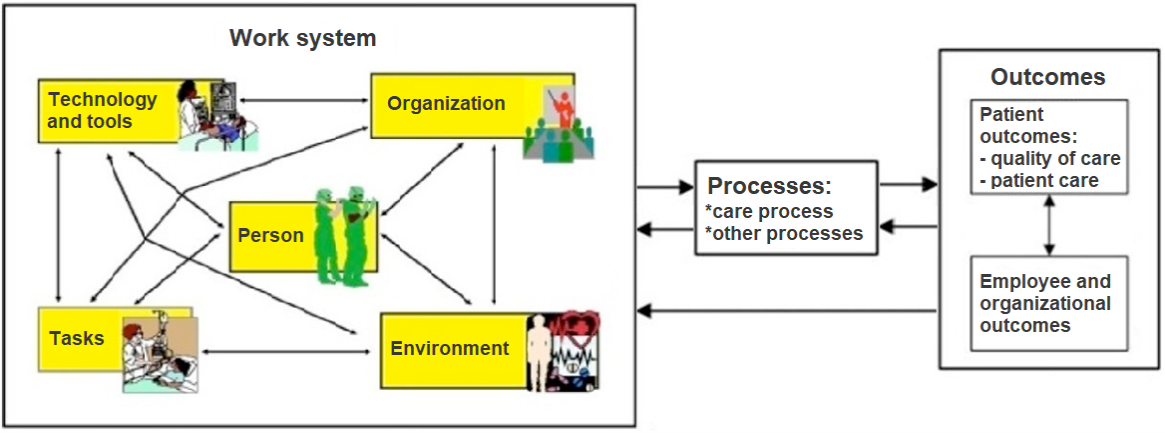
The Systems Engineering Initiative for Patient Safety (SEIPS) framework of work system, processes, and outcomes.

Anchored within the STS tradition is the SEIPS framework, illustrated in Figure 1, which applies a whole system perspective on health care [36]. The framework details elements within a work system (person, technology and tools, task, environment, and organization) that interact and are mutually interdependent. The design of the work system affects different processes (eg, clinical treatment and patient follow-up) that influence different outcomes related to the patient, employee, and organizational outcomes [36]. A core aspect of the SEIPS framework, in relation to the TELE-AMBUS project, lies in its ability to inform implementation, change management, and overall design of complex systems, thereby contributing towards prevention and avoidance of undesired outcomes across system performance and well-being in a health care context, specifically in terms of patient outcomes (mortality, complications, quality of life, and medical errors), organizational outcomes (efficiency and treatment time), and employee outcomes (well-being aspects such as job satisfaction and motivation) [37]. The feedback loops in the framework suggest that knowledge or insights into the dynamics of the model, including outcomes, can be applied to adjust and redesign the work system [37].

The outpatient model relates to the SEIPS framework (Figure 1) as follows: The specialist AWCT and primary care nurses constitute the center of the work system (person aspects), enabled or constrained by other aspects of the work system spanning both specialist and primary health care. For example, technology aspects of the work system include the telemedicine solution (used by both specialist and primary health care) and the patient journal system, that is, Distributed Information and Patient Data System (used by the specialist health care), while organizational aspects include staffing and competency levels and priority given or time allocated to outpatient wound management in everyday practices. Thus, the outpatient model and work system understanding take on an interorganizational and interprofessional form, encompassing persons involved, the technology used, the specifics of the organization, and so forth across services. This cross-sector work system influences the outpatient wound care process (middle part of SEIPS) in terms of enabling or constraining the work tasks of the wound specialist nurses (eg, evaluation of the patient’s wound, bedside teaching of primary care nurses in wound care procedure and how to use telemedicine) and the primary care nurses (eg, follow-up of wound development including the transference of pictures through the telemedicine solution). Finally, the quality of the outpatient wound care process produces organizational and individual outcomes (the right part of SEIPS) that are desirable to a larger or lesser degree. For the TELE-AMBUS project, these outcomes concern (1) the costs and benefits of the regular clinical versus outpatient treatment models and (2) changes in health care provider competence and quality across specialist and primary health care services. The latter outcome is tied to the project’s degree of success or not in transforming empirical findings into managerial and employee strategies and practices across services.

While studies using the SEIPS framework have become substantial in recent years, there is a lack of SEIPS-based intervention studies, in particular (1) studies that jointly consider physical, cognitive, and organizational HFE issues and (2) studies that apply a broad approach to outcomes linking design with patient outcomes [37]. Furthermore, the involvement of end users (patients and providers) is emphasized from a participatory perspective [37]. Finally, reviews show that with a few exceptions, there is a lack of studies analyzing health care with an HFE perspective within the Scandinavian context [37-39]. The operationalized SEIPS framework enables us to explore and analyze implementation and design aspects of the outpatient model, comprised of interconnected telemedicine and AWCT interventions embedded within existing organizations and practices across the specialist health care services, the primary health care services, and, not least, the specific context of the chronic wound patient.

The model can analytically appear daunting due to its dynamic nature, that is, in the presentation of mutually interacting work system relationships, but when purposefully operationalized, it enables the researcher to capture the intricacy of the human-technological–organizational complexity under study. In our operationalization, we applied the 5 work system dimensions of the SEIPS model, that are internal environment, external environment, individual and team, organization, and technology and tools, not only to create a mental orientation for our fieldwork (in terms of what to look for during the patient visits) but also as coding guidance when processing data in the computer-assisted qualitative data analysis software QSR NVivo (Lumivero) and Dedoose (SocioCultural Research Consultants).

### Ethical Considerations

The TELE-AMBUS project does not apply a clinical trial design and approach; the project seeks to improve the quality and holistic integration of wound diagnosis and treatment across primary and specialist health care services. This is achieved by exploring and analyzing cross-sector implementation and consequences of a new outpatient chronic wound management model comprising an ambulatory wound team and telemedicine components. In other words, the project’s purpose is to study and improve current health services across sectors, and not to procure knowledge on the patient’s health and disease.

Specifically, 1-2 researchers observe the ambulatory wound team’s consultation and interaction with municipal nursing staff and the patient. Written observational notes focus exclusively on service quality aspects, that is, no personal or sensitive information will be taken down including from patients, and no audio or visual recording will be involved during the observations. The only audio-recorded data will be semistructured interviews, involving the health care personnel, management, and information and communications technology personnel from specialist health care. While it should be noted that audio recordings could potentially contain background information that enables identification of a person, all background and other possible person-identifiable information are deleted during transcription.

Written information and consent forms about project purpose and participation, secure data processing and storage, a field for providing written consent, and so forth corresponding to the Norwegian Centre for Research Data templates, are sent to and gathered from all project participants including primary and specialist health care personnel and managers as well as patients. While no personal or sensitive information is collected from any participants including patients, informed consent is secured from each patient in advance of the ambulatory wound team’s consultation visit to allow for the researchers’ presence and observations of health care personnel during the visits. Beginning in January 2023 and in advance of the patient visits and fieldwork, the hospital partner involved in this project also sent out an informal information letter to all 6 participating municipalities. This letter describes in detail the practical aspects of the research project, including how the ambulatory wound team will operate across sectors and the researchers’ specific roles and involvement.

Based on the above premises, the TELE-AMBUS research project was accepted by the Regional Committee for Medical Research Ethics Western Norway (application ID 375986) and approved by the Norwegian Centre for Research Data (reference 236558). The project has also been agreed upon by the internal data protection representative at Stavanger University Hospital (protocol 2847-2847).

### Data Collection and Analysis

As part of this project’s qualitative longitudinal study design (discussed in the *Research Approach* section), empirical field data are gathered through observations, semistructured interviews, and informal conversations across the specialist and primary health care services. The resulting data are analyzed through QSR NVivo and Dedoose, which enables systematization and transparency in both the analysis and dissemination of results, improving the reliability and trustworthiness of the study [27,40]. As we apply these approaches to the TELE-AMBUS project, we seek to map emergent patterns and connections in the data material related to (1) the implementation and consequences of the outpatient wound management model and (2) associated improvements in health care provider competence and overall service quality across specialist and primary health care services (discussed in the *Primary and Secondary Project Objectives* section).

As part of economic evaluation (refer to the *Research Approach* section) and based on data gathered through the qualitative empirical fieldwork (as described above), costs associated with a generic pathway in the regular clinical wound model are calculated as costs at the primary health care level (eg, staff hours, wages, medical equipment, and so forth) and costs at the specialist health care level (eg, staff hours, wages, length of inpatient stay, and so forth). We do the same costing exercise for the new outpatient model comprising telemedicine and the AWCT. We conduct one-way and deterministic sensitivity analyses as well as probabilistic sensitivity analyses (if feasible) for the quantitative cost-benefit analyses. The analyses feed into the overall objective of mapping emergent patterns and connections in the data material related to the implementation and consequences of the outpatient wound management model and associated improvements in provider competence and service quality.

## Results

The project has been funded from 2021 to 2025. A series of baseline qualitative focus group interviews and semistructured interviews with project participants or stakeholders at the hospital and in the 6 municipalities have been conducted since April 2022 and concluded in January 2024. Starting November 2022, the AWCT has conducted outpatient visits and follow-up telemedicine consultations, with the latter part lasting until the wound has healed or the patient’s death (typically, patients are older adults with multimorbid conditions). The patient’s wound condition is evaluated during the initial visit and subsequent follow-up telemedicine consultations. The outpatient visits are expected to end in mid-2025. Fieldwork, including observations, semistructured interviews, and informal conversations, has been undertaken with project participants and stakeholders at the hospital and in the 6 municipalities since November 2022 and is expected to conclude in March 2025. The baseline interviews and subsequent fieldwork cover WP 2 and WP3 in the TELE-AMBUS project, focused on exploring the intervention from an STS perspective as well as documenting health economic costs and effects of implementing the intervention, respectively (Table S1 in Multimedia Appendix 1). The results of WP2 and WP3 feed into WP4 on transforming project findings into cross-sectoral managerial and employee strategies and practices. Initial empirical results from the baseline interviews were presented at the 14th Organizational Design and Management Conference [41]. In addition, 1 peer-reviewed publication has been published in the International Wound Journal [6], as part of WP1 targeting a cross-sector synthesis of existing knowledge on telemedicine and chronic wound management interventions (Table S1 in Multimedia Appendix 1).

Note that the Gantt chart provided in Table S1 in Multimedia Appendix 1 also elaborates on the roles and tasks of the project participants, such as designated researcher groups and advisory boards spanning the years and quarters of the project’s time span.

## Discussion

### Principal Findings

In terms of the TELE-AMBUS project status, findings of systematic reviews conducted in WP1 indicate that there is a pressing need for more comprehensive empirical explorations into quality improvement and integration of telemedicine in chronic wound management, including using system frameworks that can capture cross-sector system perspectives and associated implications. We specifically suggest that the design and execution of telemedicine improvement interventions and associated research projects should be conducted in close cooperation with managers and practitioners knowledgeable about barriers and opportunities that can influence the implementation of important interventions within chronic wound management [6]. The baseline results from WP2 and WP3, to be finalized this year, and the larger fieldwork data collection and analysis processes, to conclude in March 2025, are expected to reveal insights into organizational, environmental, technological, individual (personnel), and cost-effect considerations that are critical to account for in the design or redesign of telemedicine and outpatient model interventions across the primary and specialist health care sectors.

We apply the sociotechnical SEIPS framework in multiple and creative ways, that is, to design and inform our fieldwork and explore, evaluate, and redesign the outpatient wound management model and work systems across the primary and specialist health care sectors. Thus, SEIPS becomes a pedagogic tool to translate and implement project findings into practice across services. To our knowledge, such an approach has not been undertaken in telemedicine in chronic wound management literature or associated HFE research. Specifically, existing cross-sector quality improvement studies focus on identifying telemedicine-related barriers, benefits, acceptance levels, and outcome variables rather than on transforming findings into cross-sectoral managerial and employee strategies and practices and redesigning (work) systems across the primary and specialist health care sectors and services [6,42]. Thus, our approach can produce both original and novel research and theoretical results internationally. The project’s tandem exploration of the 2 quality improvement interventions within telemedicine in chronic wound management (combining telemedicine and AWCT to bring specialist and primary health care sectors closer) has, to our knowledge, not been undertaken in a Norwegian context and thus can produce novel national results within the field of telemedicine and chronic wound management.

### Strengths and Limitations

As is the case for any research project using a qualitative design approach, extrapolation and generalizability of the resulting findings can be problematic and, thus a limitation [43,44]. However, a qualitative approach offers unique, in-depth insight into the richness and complexity of the phenomenon under study. Applied to the TELE-AMBUS project, we use process and economic evaluations to capture why and how the studied intervention (outpatient model) contributed to change or not, including the associated mechanisms and outcomes at individual and organizational levels. Our primary research interest lies in knowing how the intervention works in practice across the involved organizations and influencing work system factors, hence allowing for redesigning or adjusting per the identified barriers. Thus, we address calls for whole system approaches within telemedicine in chronic wound management research and STS research (discussed in the *Introduction* and *Research Approach* sections, respectively).

### Conclusions

As noted in our systematic review [6], telemedicine interventions in chronic wound management must adopt a longitudinal and broader system perspective across primary and specialist health care services that accounts for clinical applications, team approaches, and wound management and implementation strategies. The system perspective must also consider the quality of life, the existing care system, and cost aspects. These research needs are reflected in the new wound management model explored in the TELE-AMBUS project. This includes not only insights the project produces by means of identifying sociotechnical barriers and facilitators and cost-effectiveness aspects associated with the outpatient model, in the latter case or analyses compared with a regular treatment model, but also by “thinking system redesign” including everyday health care management and professional strategies and practices across the primary and specialist health care sectors. This way of connecting system theory and empirical findings to actual practice (transformation) represents a key element and novelty of the TELE-AMBUS project with a potential impact spanning research and practice fields from HFE to eHealth and chronic wound management.

## Appendix

Multimedia Appendix 1 Telemedicine and Ambulatory Wound Care Team (TELE-AMBUS) Gantt chart from 2021 to 2025, detailing main tasks and participants across work packages and years.

## Abbreviations

AWCT: ambulatory wound care team
HFE: human factors and ergonomics
ICD-10: International Classification of Diseases, Tenth Revision
STS: sociotechnical system
SEIPS: Systems Engineering Initiative for Patient Safety
TELE-AMBUS: Telemedicine and Ambulatory Wound Care Team
WDC: Wound Diagnostic Centre
WP: work package

## Data Availability

The economic datasets generated and analyzed during this study will be limitedly accessible in the Norwegian Centre for Research Data repository once the embargo is lifted on January 1, 2027.
