# Supplementary material for: A Combined Telemedicine and Ambulatory Wound Care Team Intervention for Improving Cross-Sector Outpatient Chronic Wound Management: Protocol for the Mixed Methods TELE-AMBUS Research Project
Source: JMIR Res Protoc. 2024 Nov 4;13:e55502. doi: 10.2196/55502 (PMC11574493; doi:10.2196/55502)
Supplement: Multimedia Appendix 1 [file resprot_v13i1e55502_app1.docx]

**Table S1.** Telemedicine and Ambulatory Wound Care Team (TELE-AMBUS) Gantt chart from 2021 to 2025, detailing main tasks and participants across work packages and years.

| WP^a^ | Main task details | Participants and roles | Supporting activities | 2021 | | 2022 | | | | 2023 | | | | 2024 | | | | 2025 | | |
| --- | --- | --- | --- | --- | --- | --- | --- | --- | --- | --- | --- | --- | --- | --- | --- | --- | --- | --- | --- | --- |
|  |  |  |  | Q^b^3 | Q4 | Q1 | Q2 | Q3 | Q4 | Q1 | Q2 | Q3 | Q4 | Q1 | Q2 | Q3 | Q4 | Q1 | Q2 | Q3 |
|  |  |  |  |  |  |  |  |  |  |  |  |  |  |  |  |  |  |  |  |  |
| WP0 | - Task 1: Project management and communication across partners, countries, sectors, and so forth | The NORCE^d^ project manager conducts the task | NORCE project support, FAST^d^ team, etc | ✓ | ✓ | ✓ | ✓ | ✓ | ✓ | ✓ | ✓ | ✓ | ✓ | ✓ | ✓ | ✓ | ✓ | ✓ | ✓ | ✓ |
| WP1 | - Task 1: Initial systematic mapping review - Task 2: Cross-sector systematic literature review and STARLITE^e^ - Task 3: Synthesis and literature-informed SEIPS^f^ framework | CRG^g^ conducts the tasks; EIRG^h^ and ISAB^i^ provide critical input on tasks | Initial and end-of-WP digital meetings with EIRG and ISAB | ✓ | ✓ |  |  |  |  |  |  |  |  |  |  |  |  |  |  |  |
| WP2 | - Task 1: Based on WP1 and SEIPS, exploring the outpatient model, identifying barriers, facilitators, and outcomes - Task 2: Through findings and partner inputs, redesigning the outpatient model using the SEIPS framework | CRG conducts the tasks; all other partners provide critical input on SEIPS framework, field design, and findings | Initial, middle, and end-of-WP digital meetings and seminars involving EIRG, ISAB, CPAB^j^, RMU^k^, and PRO^l^ |  |  | ✓ | ✓ | ✓ | ✓ | ✓ | ✓ | ✓ | ✓ | ✓ | ✓ | ✓ | ✓ | ✓ |  |  |
| WP3 | - Task 1: Cost-effectiveness assessments of the outpatient model compared to the traditional service delivery model | CRG conducts the tasks; EIRG and ISAB provide critical input on task | Initial, middle, and end-of-WP digital meetings with EIRG and ISAB |  |  | ✓ | ✓ | ✓ | ✓ | ✓ | ✓ | ✓ | ✓ | ✓ | ✓ | ✓ | ✓ | ✓ |  |  |
| WP4 | - Task 1: Based on WP1-3 and SEIPS, transforming knowledge into cross-sector management and practices, including a redesign of the current outpatient model - Task 2: Main dissemination of project deliverables and outputs | CRG and EIRG conduct the tasks, merging national and international insights and experiences | Initial, middle, and end-of-WP digital meetings and seminars involving EIRG, ISAB, CPAB, RMU, and PRO |  |  |  |  |  |  |  |  |  |  |  |  | ✓ | ✓ | ✓ | ✓ | ✓ |

^a^WP: work package.

^b^Q: quarter.

^c^NORCE: Norwegian Research Centre.

^d^FAST: Research support at NORCE.

^e^STARLITE: Standards for Reporting Literature searches.

^f^SEIPS: Systems Engineering Initiative for Patient Safety.

^g^CRG: core research group.

^h^EIRG: extended international researcher group.

^i^ISAB: International Scientific Advisory Board.

^j^CPAB: Clinical Practice Advisory Board.

^k^RMU: regional municipalities.

^l^PRO: Patient Representative Organization.
